# Is cognitive behaviour therapy an effective option for women who have troublesome menopausal symptoms?

**DOI:** 10.1111/bjhp.12543

**Published:** 2021-06-08

**Authors:** Myra S. Hunter, Joseph Chilcot

**Affiliations:** ^1^ Institute of Psychiatry, Psychology and Neuroscience King’s College London UK

**Statement of contribution**

***What*** ***is known already on this subject?***

- Vasomotor symptoms (VMS) are commonly experienced by women during the menopause transition and can impact on quality of life.
- Hormone therapy is an effective treatment but is not recommended for some women, and there is a need for safe and effective non‐medical interventions.

***What*** ***this study adds?***

- This editorial describes the development and evaluation of a cognitive behavioural intervention for women with problematic VMS.
- A series of clinical trials demonstrate the effectiveness of the CBT delivered in various formats to women, and men, in different contexts.

Hormonal and menstrual changes and hot flushes typically occur during the menopausal transition, but women’s experience of the menopause, as a whole, is very much influenced by psychological and social factors, such as symptom appraisal, coping strategies, past experience, lifestyle, social and cultural meanings of menopause, and a woman’s social and material circumstances (Hunter & Rendall, 2007). It is awareness of these – often neglected – influences that provided the groundwork for the development of non‐medical interventions as alternatives and/or adjuncts to hormone therapy (HT). While HT is the recommended medical treatment for menopausal symptoms (National Institute for Health and Care Excellence (NICE), 2015), it is not indicated for all women, such as those with a history of hormone‐dependent cancers or venous thromboembolism (NICE, 2015; Szabo, Marino, & Hickey, 2019). Moreover, many women prefer non‐hormonal and/or non‐medical treatments (Gentry‐Maharaj et al., 2015; Hickey, Szabo, & Hunter, 2017). This editorial outlines the evaluation and implementation of CBT (MENOS protocol) for women with problematic menopausal symptoms (Ayers, Mann, & Hunter, 2011; Mann, Smith, Hellier, & Hunter, 2011).

Hot flushes and night sweats, or vasomotor symptoms (VMS), are the main physical symptoms attributable to the menopause, affecting approximately 80% of women, and the main reason that women seek treatment (Guthrie, Dennerstein, Taffe, & Donnelly, 2003). Temperature regulation is affected by reducing levels of oestrogen and is associated with changes in central nervous system neurotransmitters, causing heat, sweating, redness and sometimes shivering, with varied duration and severity (Archer et al., 2011). An estimated 25–30% of women report problematic VMS (i.e., flushes that impact on daily life). Interestingly, it is the extent to which the VMS are perceived as problematic, rather than their frequency, that is associated with quality of life (QOL) and help‐seeking behaviours (Ayers & Hunter, 2013; Hunter, Nuttall, & Fenlon, 2019), which consequently is the main outcome that we seek to change.

There are bi‐directional relationships between VMS and mood, and both can lead to sleep problems and reduced QOL (Maki et al., 2019). Having persistent and/or troublesome VMS and sleep problems can make women feel stressed and irritable, which in turn can exacerbate menopausal symptoms. An estimated 9–10% of women report an increase in psychological symptoms, including depressed mood and/or anxiety, during the menopause transition (Almeida et al., 2016; Mishra & Kuh, 2012), which tends to be relatively transient, and is associated with troublesome VMS, current stress, low self‐esteem, and hormone fluctuations (Arnot, Emmott, & Mace, 2021; Campbell, Dennerstein, Finch, & Szoeke, 2017; Willi, Süss, Grub, & Ehlert, 2021). Longer duration of VMS has been linked with higher levels of perceived stress (Avis et al., 2015); acute psychological stress has been shown to provoke VMS (Swartzman, Edelberg, & Kemmann, 1990); there is also some evidence of altered physiological stress responsivity in women with VMS (Nathan et al., 2020). Several factors such as poor health, past depression, low income, perceived stress, attitudes to ageing and menopause, and early life circumstances are associated with both troublesome VMS and low mood (Alexander et al., 2007).

Cross‐cultural studies suggest variations in perceptions and experience of menopause in women from different ethnic origins and living in different countries (Avis & Crawford, 2008). For example, in Western countries women tend to have more negative perceptions of the menopause and ageing and report more VMS, than those living in India, Japan, and China (Freeman & Sherif, 2007). The results of a multicentre US study (Avis et al., 2001) found different patterns of menopausal symptoms evident amongst women of different ethnicities. African‐American women reported high frequency of hot flushes, East Asian women reported low frequency and Caucasian women were found to report more psychological and somatic symptoms than any other ethnic groups. There is also variation between women within ethnic groups, as well as variation across cohorts and generations (Utz, 2011).

Attitudes and beliefs about menopause can influence the type and number of physical and emotional changes (e.g., visual problems, mood swings, high blood pressure) that women attribute to it (Hunter, Gupta, Papitsch‐Clarke, & Sturdee, 2009; Hunter & O’Dea, 2001). The attribution of symptoms to menopause is important, since, apart from VMS, other ‘menopausal symptoms’ may well have other causes or are likely to interact with other factors that are common during midlife, such as work demands, caring roles, and accumulated stresses. There is also evidence that negative attitudes and expectations, held before the menopause, predict distress and symptom experience during the menopause (Ayers, Forshaw, & Hunter, 2010), and that beliefs and attitudes towards menopause tend to be more positive amongst older, postmenopausal women who tend to report more neutral or positive changes, for example, not having menstrual periods (Brown, Brown, Judd, & Bryant, 2018; Hvas, 2006; Smith, Mann, Mirza, & Hunter, 2011). Social attitudes may in turn influence an individual woman’s views about menopause, as well as her appraisal of VMS (Rendall, Simmons, & Hunter, 2008). We have found that negative beliefs and appraisals of VMS and behavioural avoidance (e.g., avoiding social situations) were significantly associated with more problematic VMS (Hunter, Ayers, & Smith, 2011; Rendall et al., 2008; Reynolds, 2000). The three main types of negative beliefs and appraisals of VMS are:

1. Beliefs about hot flushes in a social context, ‘they are embarrassing and shameful’,
2. Perceived control over hot flushes, ‘They will never end, I can’t cope’, and
3. Beliefs about night sweats and sleep, ‘If I wake I’ll never get back to sleep’, ‘if I have night sweats I’ll feel terrible the next day’.

Women often report that VMS symptoms are more difficult to deal with while at work, due to embarrassment and worry about the reactions of others (Griffiths & Hunter, 2015). Not surprisingly, if women think that they are viewed as ‘unattractive, old, or useless’ – thoughts which are associated with unduly negative stereotypes about menopause in general – they are more likely to feel distressed. Having to hide signs of menopause is often mentioned as an additional source of anxiety when at work. Certain situations at work can precipitate or exacerbate hot flushes such as work stress, high visibility tasks such as presentations, uniforms, and hot and poorly ventilated environments (Hardy, Griffiths, Norton, & Hunter, 2018; Hardy, Thorne, Griffiths, & Hunter, 2018).

Based on this research, Hunter and Mann (2010) developed a theoretical model of VMS that draws upon symptom perception, self‐regulation, and cognitive behavioural theories to explain women’s perceptions, appraisals, and reactions to VMS symptoms (Figure 1). The model includes the influence of concurrent stress, mood, negative beliefs, and behaviours. Internal and external factors, such as anxiety, spicy food, and alcohol, can trigger hot flushes, while attentional focus on bodily sensations (somatic amplification) is associated with VMS reporting. Cognitive reactions can exacerbate distress and VMS, for example, negative beliefs about menopause and VMS (relating to social embarrassment, disgust, feeling out of control and worry about sleep) and behavioural reactions, such as avoiding social situations, are associated with more problematic VMS.

**Figure 1:**
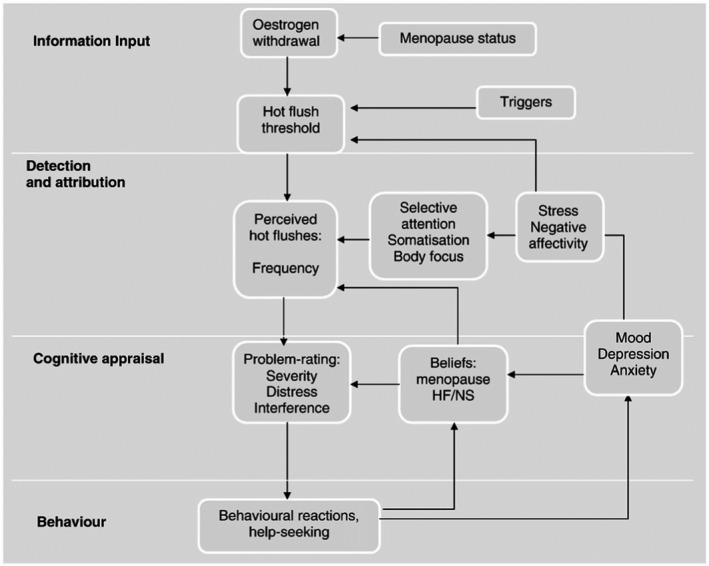
A cognitive model of hot flushes and night sweats (Hunter & Mann, 2010) reproduced with kind permission of Elsevier.

We tested the model by investigating the relationships between perceived stress, mood, VMS beliefs and subjective and physiological measures of menopausal VMS (Hunter & Chilcot, 2013). Over half (53.2%) of the variance in VMS problem rating could be explained; with stress, anxiety, and somatic amplification predicting VMS problem rating, but only via their impact on VMS beliefs. These findings support the CBT model at the level of symptom perception and cognitive appraisal.

The MENOS CBT treatment protocol was based on this model, being interactive and educational, with a focus on helping people to reduce stress, to manage hot flushes and night sweats, and improve sleep (Ayers et al., 2011; Mann et al., 2011). Relationships between physical symptoms, cognitions (thoughts and beliefs), feelings and behavioural reactions are examined, using educational PowerPoint slides, group discussion, handouts, and homework to be worked on between sessions. Negative beliefs about hot flushes in social contexts, such as ‘everyone is looking at me’, can lead to feelings of shame and embarrassment and increased physiological arousal, which in turn increases hot flush intensity. Women might then wish to leave or avoid situations believing that they are unable to cope. The intervention aims to counter these negative cycles using a range of strategies to help women to deal with stress, VMS, and sleep problems. In summary, the sessions include:

- Information about menopause and VMS with physiological explanation and introduction to the cognitive behavioural model
- Identifying individual goals
- Monitoring and modifying hot flush triggers or precipitants
- CBT to reduce stress and improve well‐being, including diaphragmatic breathing exercise
- Exploring the impact of unhelpful cognitions (thoughts, beliefs, and attitudes) relating to menopause, ageing, and VMS, and encouraging supportive alternatives
- Encouraging helpful behavioural strategies, for example, reducing avoidance, pacing activities
- Information and CBT strategies for managing night sweats and sleep
- Maintenance plans.

The group CBT treatment targets are worked on over 4 or 6 weekly sessions comprising 8 hr in total and were delivered by a clinical health psychologist in the MENOS trials. Self‐help CBT involves a booklet and audio with up to 1.5 hr of telephone guidance, with the same content as group CBT, followed over 4 weeks. Goals are agreed at the outset, and individualized homework is carried out between sessions. The final session includes a review and maintenance plan to encourage the continuation of the strategies that the women found helpful.

Clinical trials assessing CBT have tended to use the Hot Flush Problem‐Rating as the primary outcome measure and hot flush frequency, mood, sleep, and QOL as secondary outcomes. The Hot Flush Rating Scale is a short scale which measures hot flush and night sweat frequency and ‘problem rating’ (three items including distress, extent to which VMS are a problem and interference with daily routine; Hunter & Liao, 1995; Hunter et al., 2019). Hot flush problem rating is associated with work and social adjustment, QOL, and use of health services. Sternal skin conductance, a psychophysiological measure of VMS, is included in MENOS1 (Mann et al., 2012) and MENOS2 (Ayers, Smith, Hellier, Mann, & Hunter, 2012) trials, although the concordance between subjective and physiological measures is relatively low (Mann & Hunter, 2011).

The trials, including 1,166 women in total, are shown in Table 1 and consistently show moderate to large effect sizes for CBT compared with usual care or wait list control. CBT significantly reduced the impact (problem rating) of VMS, compared with a wait control, in MENOS 1 and 2 trials (Ayers et al., 2012; Mann et al., 2012), including patients with breast cancer and well women, respectively, by an average of 50%, with improvements maintained at 26‐week follow‐up. Comparison of group CBT with self‐help CBT (MENOS2) suggested that both forms of CBT were equally effective in reducing problematic VMS; both improved night sweat frequency and mood, but group CBT resulted in more improvements to QOL compared with self‐help CBT. CBT reduced subjective frequency of night sweats by an average of 39% and reduced objectively measured hot flushes (sternal skin conductance monitoring) in well women, but not in patients with breast cancer (Stefanopoulou & Hunter, 2013). This may be due to differences in the nature of menopausal symptoms in patients with breast cancer, since they may be influenced by cancer treatments.

**Table 1:** Randomized controlled trials of CBT using the MENOS protocol and the Problem rating of the Hot Flush Rating Scale (HFRS) as the prime outcome measure with effect sizes (Cohen *d*; 0.2 (small), 0.5 (moderate), and 0.8 (large)

Trial name	Design	Outcome measure	Treatment *N* per arm	*N*	Results effect sizes post randomization^a^	Follow up effect sizes^a^
MENOS1 Mann et al *Lancet Oncology* 2012	RCT Group CBT vs usual care for breast cancer patients with VMS	HFRS at 9 and 26 weeks	Six 90 min Group CBT sessions *n* = 47 Usual care *n* = 49	96	Group CBT **0.75** at 9 weeks, *p* < .001	Group CBT **0.79** at 26 weeks, *p* < .001
MENOS2 Ayers et al *Menopause* 2012	RCT Group CBT vs Self‐Help CBT vs no treatment control (NTC) for well women with VMS	HFRS 6 and 26 weeks	Four weekly 2‐hr Group CBT sessions *n* = 48 vs self‐help *n* = 47 vs NTC *n* = 45	140	Group CBT **0.86** Self‐help CBT **0.88** at 6 weeks, *p* < .001	Group CBT **0.54** at 26 weeks, *p* < .001 Self‐help CBT **0.50** at 26 weeks, *p* < .005
EVA Duijts et al *J Clin* *Oncology* 2012	RCT Group CBT + exercise (PE), vs CBT vs PE vs Usual care for breast cancer patients with VMS	HFRS 12 weeks and 6 months	Six weekly 90‐min Group CBT+ PE *n* = 106, CBT *n* = 109, PE *n* = 104, Usual care *n* = 103	422	CBT **0.73** and CBT+ PE **0.84** at 12 weeks, *p* < .001, PE 0.12 ns	CBT **0.62** and CBT+ PE **0.55** at 6 months *p* < .001, PE 0.03 ns
MENOS@Work Hardy et al *Menopause* 2018	RCT unguided Self‐Help CBT vs Wait list control for women with VMS at work	HFRS 6 and 20 weeks	Self‐help CBT booklet over 4 weeks *n* = 60, Wait list *n* = 64	124	Self‐help CBT **0.77** at 6 weeks, *p* < .001	Self‐help CBT 0.**56** at 20 weeks, *p* < .01
EVA online iCBT Atema et al *J Clin* *Oncology* 2019	RCT Online CBT therapist guided vs unguided vs usual care for breast cancer patients with VMS	HFRS 10 weeks and 24 weeks	Guided CBT (iCBT) over 6 weeks *n* = 85, unguided iCBT *n* = 85, Usual care *n* = 84	254	Guided **0.63**, unguided **0.56**, iCBT at 10 weeks, *p* < .001	Guided **0.48**, unguided **0.40** iCBT groups at 24 weeks, *p* < .01
MENOS4 Fenlon et al *Psycho‐oncology* 2020	RCT Group CBT delivered by breast cancer nurses to patients with VMS vs Usual care	HFRS 9 and 26 weeks	Six weekly 90‐min group CBT sessions *n* = 63, usual care = 67	130	Group CBT **0.94** at 9 weeks, *p* < .0001	Group CBT **1.01** at 26 weeks, *p* < .01

^a^ Effect sizes calculated using the adjusted mean difference/baseline pooled standard deviation.

Using a single group design, we found that self‐help CBT offered to women with minimal telephone guidance (MENOS3) was effective at comparable levels found in the MENOS 1 and MENOS2 trials (Stefanopoulou & Hunter, 2014). Telephone‐based CBT has also been shown to reduce sleep problems and insomnia reported by women during the menopausal transition and postmenopause (McCurry et al., 2016).

In a Dutch study (EVA, Duijts et al., 2012), patients with breast cancer who were premenopausal when diagnosed showed a significant decrease in hot flush problem rating following group CBT compared with physical exercise or usual care at post‐treatment and 6‐month follow‐up. In order to increase accessibility to CBT for this population, we investigated the efficacy of Internet‐delivered CBT (iCBT), with and without therapist support, adapted from the MENOS protocols (Atema et al., 2019). Women in the guided iCBT arm received additional support by telephone and weekly online feedback from a trained psychologist or medical social worker. Compliance rates were high. Compared with women in the control group, those in the guided and self‐managed iCBT groups reported a significantly greater decrease in the perceived impact of VMS and improvement in sleep quality. At 24‐week follow‐up, the effects remained significant, and both intervention groups demonstrated a significant reduction in VMS frequency, as well as problem rating. iCBT was effective with and without therapist support, but the improvement was greater with some guidance.

In MENOS4, we tested whether breast cancer nurses can be trained to deliver Group CBT effectively across six oncology centres in England (MENOS4; Fenlon et al., 2020). The nurses were trained by a clinical health psychologist and two CBT groups carried out in each centre. There was a significant (46%) reduction in the mean VMS problem rating score from randomization to 26 weeks in the CBT arm compared with a 15% reduction in the usual care arm. Secondary outcomes, including frequency of VMS, sleep, anxiety, and depression all improved significantly. These results suggest that specialist nurses can be trained to deliver CBT effectively for the alleviation of troublesome menopausal VMS in women following breast cancer in a clinical setting.

We conducted the Menos@work RCT to evaluate a brief self‐help booklet version of the CBT with women working in eight UK organizations, who were experiencing problematic VMS (Hardy, Griffiths, et al., 2018; Hardy, Thorne, et al., 2018). This brief intervention, delivered without any clinical input, significantly reduced VMS frequency and problem‐ratings, and improved sleep quality at 6‐ and 20‐week follow‐up, compared with a waitlist control group. Measures of work and social adjustment and ‘presenteeism’ – being at work and having difficulty coping with symptoms – also significantly improved for women who had had CBT, compared with those who did not.

Additional studies have addressed the question of how does CBT work and for whom (i.e., moderators and mediators of the treatment effects). With regard to moderators, in MENOS2 CBT was effective regardless of age, body mass index, menopause status, or psychological factors at baseline; in other words, the effect was approximately equal for all participating women (Norton, Chilcot, & Hunter, 2014). For patients with breast cancer (MENOS1), CBT was effective at reducing VMS problem rating regardless of age, BMI, menopausal status at time of diagnosis, or type of cancer treatment. The treatment effect was significantly greater in women not receiving chemotherapy (although it was still significantly effective for those who had had chemotherapy), those with higher levels of psychological distress at baseline and for non‐white women (Chilcot, Norton, & Hunter, 2014). These findings suggest that CBT is widely applicable for women having problematic HFNS, regardless of sociodemographic or health‐related factors.

Secondary mediation analyses of MENOS 1 showed that CBT appeared to work by changing VMS beliefs, that is, cognitive appraisal, and by improving mood and sleep (Chilcot et al., 2014). For women going through the menopause transition (MENOS2), the effect of CBT on VMS problem rating was mediated by changes in cognitions (beliefs about coping/control of hot flushes, beliefs about night sweats and sleep) but not by changes in mood (Norton et al., 2014). In subsequent studies (Menos@work, EVA online and Menos4), significant changes in beliefs and behaviours were also evident. As was the case for MENOS1 and 2, the development of healthier beliefs relating to social impact of VMS, perceived control and beliefs about the impact of night sweats on sleep, mediated treatment effects in Eva online using iCBT (Atema et al., 2019). These results support the CBT model and suggest that the CBT works mainly by changing the cognitive appraisal of VMS.

Qualitative interviews with participants at the end of MENOS1 and 2 trials were conducted to explore women’s views of the treatment (Balabanovic, Ayers, & Hunter, 2012, 2013). Women reported increased confidence and ability to cope with hot flushes and night sweats; key factors mentioned were acceptance and a restored sense of control (experienced on a number of different levels and often facilitated by calm breathing). Many women reported that they attended differently to their hot flushes; for example, they may have had VMS but did not notice them. Perhaps reflecting the skills learned, the beneficial effects of the treatment, in some cases, extended beyond management of menopausal symptoms. Women also found the group context helpful in terms of normalizing their experiences, motivating them in homework tasks, and providing support.

CBT for VMS has also been evaluated for women who have VMS and depression. Conklin et al. (2020) carried out a pilot, single group study using the MENOS protocol for group CBT for 59 women with problematic VMS plus a diagnosis of mood disorders (48 had major depression; 11 bipolar disorder). Pre–post analysis revealed significant improvement for VMS problem rating, mood, anxiety, and QOL. There was good compliance and CBT intervention was considered to be acceptable and feasible for women with psychological disorders. Using a different protocol (CBT‐Meno), Green et al. (2019) evaluated a 12‐session CBT group‐based treatment for women who had both problematic VMS and moderate to severe depressed mood in an exploratory trial with significant reductions in VMS interference and bother, and in self‐reported depressive symptoms at 3‐month follow‐up.

VMS can also affect men. Androgen deprivation therapy (ADT) is a common hormonal treatment for prostate cancer; however, it may be associated with troublesome side effects that include VMS which can be frequent and severe and impact on QOL. In an exploratory trial (MANCAN), we found that a self‐help CBT (booklet adapted for men) significantly reduced VMS in men with prostate cancer when delivered by a clinical health psychologist, compared with usual care (Stefanopoulou, Yousaf, Grunfeld, & Hunter, 2015); the improvements were maintained but group differences were not significant at 32 weeks. A multicentre trial is currently in progress that includes more support and a booster group session.

We have worked with community groups supporting black and minority ethnic women through the menopause and found that including the CBT programme in a 12‐week course reduced women’s reports of VMS (Bellott, Rouse, & Hunter, 2018). We are also working with charities such as Maggie’s cancer charity and Turning Point, a charity supporting people with a range of health problems, to increase access to the treatment via training and online services. Self‐help books (Hunter & Smith 2020, 2021), and a group CBT training manual for health professionals (Hunter & Smith, 2015), have been published, so that women can use the self‐help CBT themselves and health professionals can offer group CBT for women with problematic VMS. Annual training for health professionals is also available via the British Menopause Society (thebms.org.uk).

### Conclusions

The MENOS CBT protocol is a brief therapy (4–6 sessions) that is theory and evidence‐based; it is acceptable to women and effectively reduces the impact of VMS, improves sleep, and has benefits to QOL. CBT has been found to be consistently effective when delivered in groups, self‐help book, and online formats (with or without additional support). The MENOS CBT protocol has been recommended as an effective non‐hormonal treatment for VMS by The North American Menopause Society (2015), and in clinical practice statements (Hickey et al., 2017; Woyka, 2020); and for the treatment of anxiety and depression for women during the menopause transition and postmenopause (NICE, 2015).

## Conflict of interest

The authors reports no conflict of interest, but MH is author of three books that are referenced (Hunter & Smith, 2015, 2020, 2021). The authors alone are responsible for the content and writing of the paper.

## Author contribution

Myra S Hunter (Conceptualization; Writing – original draft; Writing – review & editing) Joseph Chilcot (Conceptualization; Writing – review & editing).
